# Three new species of *Elachista* Treitschke, 1833 (Lepidoptera, Elachistidae, Elachistinae) from Kenya and Tanzania

**DOI:** 10.3897/zookeys.1285.194271

**Published:** 2026-07-21

**Authors:** Virginijus Sruoga, Jurate De Prins

**Affiliations:** 1 Institute of Biosciences, Life Sciences Center, Vilnius University, Saulėtekio Ave. 7, LT-10257 Vilnius, Lithuania Institute of Biosciences, Life Sciences Center, Vilnius University Vilnius Lithuania; 2 National Research Collections Australia, Commonwealth Scientific and Industrial Research Organisation, Canberra, Australia National Research Collections Australia, Commonwealth Scientific and Industrial Research Organisation Canberra Australia

**Keywords:** Microlepidoptera, mining moths, morphology, taxonomy, sub-Saharan Africa

## Abstract

Three species of *Elachista* Treitschke, 1833 are described as new: *E. agassizi***sp. nov**., *E. knudlarseni***sp. nov**., and *E. riftella***sp. nov**. The new species are diagnosed and illustrated with photographs of the adults and male genitalia. A checklist of 25 *Elachista* species known in sub-Saharan Africa is included.

## Introduction

*Elachista* Treitschke, 1833 is the largest genus within the subfamily Elachistinae (Gelechioidea, Elachistidae), comprising 705 described species prior to the present contribution (Lees and Minet 2022; Kaila et al. 2025; Sruoga and Kaila 2026; Nel et al. 2026). The most recent and comprehensive accounts of the genus were provided by Kaila (1999, 2011), and subsequent systematic data and taxonomic changes were later integrated into the World Catalogue of Elachistinae (Kaila 2019). Although based on current knowledge, the genus appears to be most diverse in the Holarctic region, it is also represented throughout the tropics, including Africa (De Prins and De Prins 2011–2026), where the fauna remains poorly documented and taxonomically challenging due to historically sparse collecting.

The taxonomic study of *Elachista* in sub-Saharan Africa has remained fragmentary throughout its history of investigation. The taxonomic study of the genus in the region dates to the descriptions of *Elachista trifasciata* (Wollaston, 1879) from Saint Helena (Wollaston 1879) and *E. crocogastra* Meyrick, 1908 from South Africa (Meyrick 1908). Over the following 24 years, Edward Meyrick described 11 additional species—primarily from South Africa (Meyrick 1909, 1911, 1913, 1914a, 1920, 1921, 1926), with others from Malawi (Meyrick 1914b), Ethiopia, and Uganda (Meyrick 1932a, 1932b). Later, one species, *Elachista merinaella* (Viette, 1956) was described from Madagascar (Paulian and Viette 1956). Descriptions of new species resumed only after about 50-year hiatus, with four species from Kenya (Sruoga and De Prins 2009), one species from Cameroon (Sruoga and De Prins 2011), and a further four species from Ethiopia, Tanzania, and Kenya (Sruoga and Kaila 2026). Including these, only 22 species currently assigned to *Elachista* were known from sub-Saharan Africa prior to the present study.

## Material and methods

Adult specimens were examined externally using a Nikon SMZ445 stereomicroscope. The genitalia were prepared following the standard method described by Robinson (1976) and Traugott-Olsen and Nielsen (1977). The male genital capsule was stained with fuchsin, the abdominal pelt, and the female genitalia with chlorazol black (Direct Black 38/Azo Black). The genital morphology was examined using a Leica DM6 B microscope. The photographs of adults were taken with a Canon EOS 80D camera fitted with a Canon MP-E 65 mm macro lens, mounted on a macro rail (MJKZZ Qool Rail). Genitalia photographs were taken with a Leica DM6 B microscope and a Leica K3C digital camera. Zerene Stacker v. 1.0, with a retouch function, was used for image stacking. All images were optimised and grouped into figures using Adobe Photoshop CC 2019.

The descriptive terminology of morphological structures follows Traugott-Olsen and Nielsen (1977), with some modifications by Kaila (1997, 1999, 2011). The forewing length was measured along the costa from the wing base to the apex of the terminal fringe scales with an ocular micrometre. The head width was measured between the inner edges of the antennal bases. A comparison of the length of the phallus in relation to the valva was measured as the longest line from the base of the sacculus to the apex of the cucullus.

The type specimens of the new species are deposited in the research collection of David J. L. Agassiz, Weston-super-Mare, United Kingdom (**DJLA**), and the Zoological Museum of Vilnius University, Lithuania (**VU**).

### Abbreviations for depositories

**DJLA** The research collection of David J. L. Agassiz, Weston-super-Mare, United Kingdom

**MNHN** Muséum national d’Histoire naturelle, Paris, France

**MZH** Finnish Museum of Natural History, Helsinki, Finland

**NHMUK** Natural History Museum, London, United Kingdom

**RMCA** Royal Museum for Central Africa, Tervuren, Belgium

**SAMC** Iziko South African Museum, Cape Town, Republic of South Africa

**TMSA** Ditsong National Museum of Natural History (formerly Transvaal Museum) Pretoria, Republic of South Africa

**VU** Zoological Museum of Vilnius University, Lithuania

**ZMUC** Zoological Museum of the University of Copenhagen, Denmark

## Taxonomy

### Elachista agassizi sp. nov.

Taxon classificationAnimaliaChordarialesChordariaceae

0EB214B9-D5BB-51B4-A008-C0235C37EDC7

https://zoobank.org/5413FFD1-474A-4B77-A793-635A31957877

Figs 1, 2

#### Material examined.

***Holotype***. Kenya • ♂; Rift Valley, Mpala Res. C.; 1720 m; 0.2908°N, 36.8977°E; 27. Nov. 2008; D. Agassiz, L. Aarvik & A. J. Kingston leg.; gen. prep. VS637; DJLA. ***Paratypes***. Kenya • 1♂; same data except date; 26. Nov. 2008; • 1♂; same data as holotype; gen. prep. VS588; • 1♂; Central Naro Moru; 1960 m; 0.1513°S, 37.0111°E; 1. Dec. 2008; D. Agassiz, L. Aarvik & A. J. Kingston leg.; DJLA.

#### Diagnosis.

*Elachista agassizi* is easily distinguished from all other Elachistinae by the uniquely sclerotised latero-basal margin of the uncus lobe with its highly modified scales, and by the complete absence of the gnathos.

#### Description.

**Figure 1.**
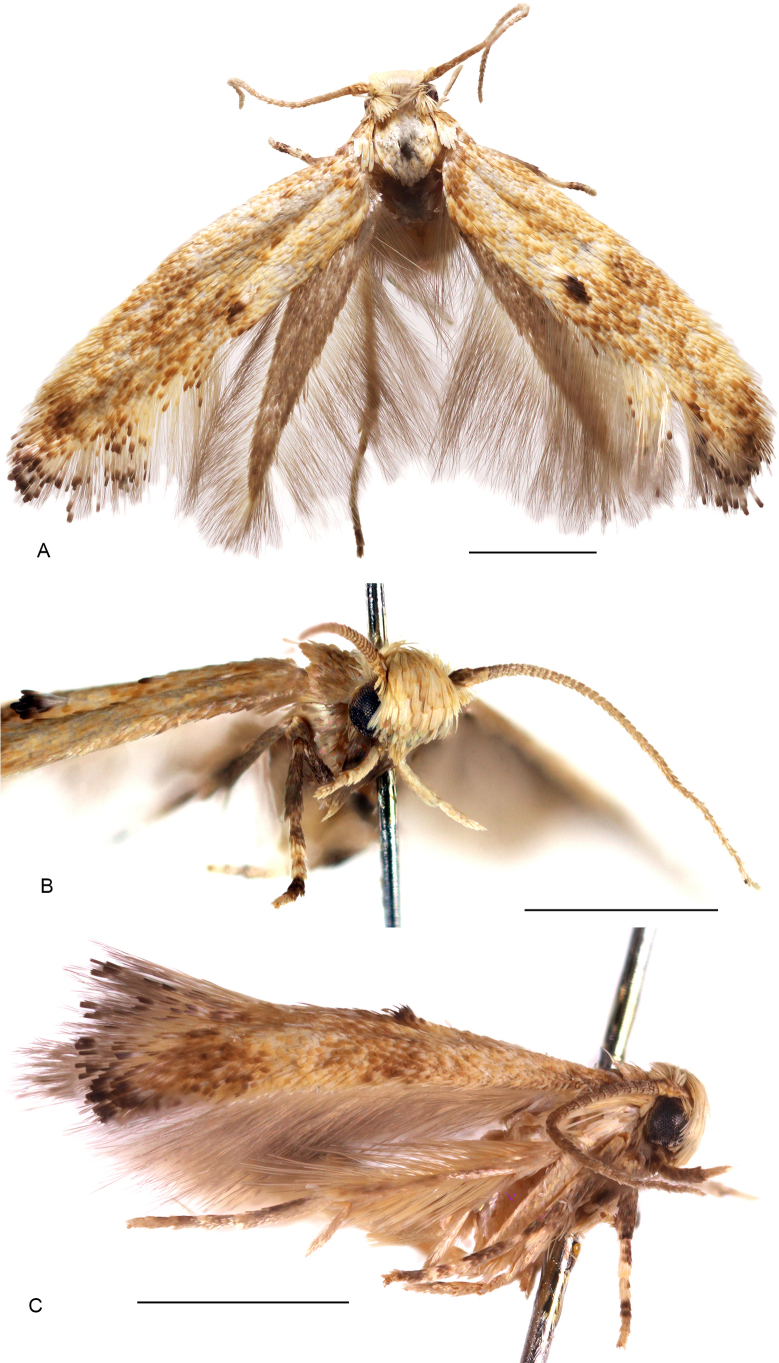
*Elachista agassizi* sp. nov., adult male. **A**. Holotype; **B**. Head, fronto-lateral view, paratype; **C**. Lateral view, paratype. Scale bars: 1 mm.

**Male**. (Fig. 1). Forewing length 3.3–3.8 mm; wingspan 7.4–8.2 mm (*N = 4*). ***Head***: pale yellowish ochreous, vertex with few darker tipped scales, neck tuft pale yellowish ochreous; labial palpus slightly upcurved, about 1.3× as long as width of head, pale yellowish ochreous, second segment dark ochreous below; antenna 0.6× as long as forewing, pecten pale yellowish ochreous, flagellum dark ochreous. ***Thorax*** pale yellowish ochreous; tegula and forewing ground colour pale yellowish ochreous, with admixture of irregular darker scales; brown-black, slightly raised scales forming two small, transversally arranged spots just before middle of forewing; subapical dorsal fringe creamy white, apical fringe brownish grey, fringe line brownish black. Hindwing brown-grey, fringe scales shortly yellowish along outer margin, otherwise pale brownish grey.

**Female**. Unknown.

**Figure 2.**
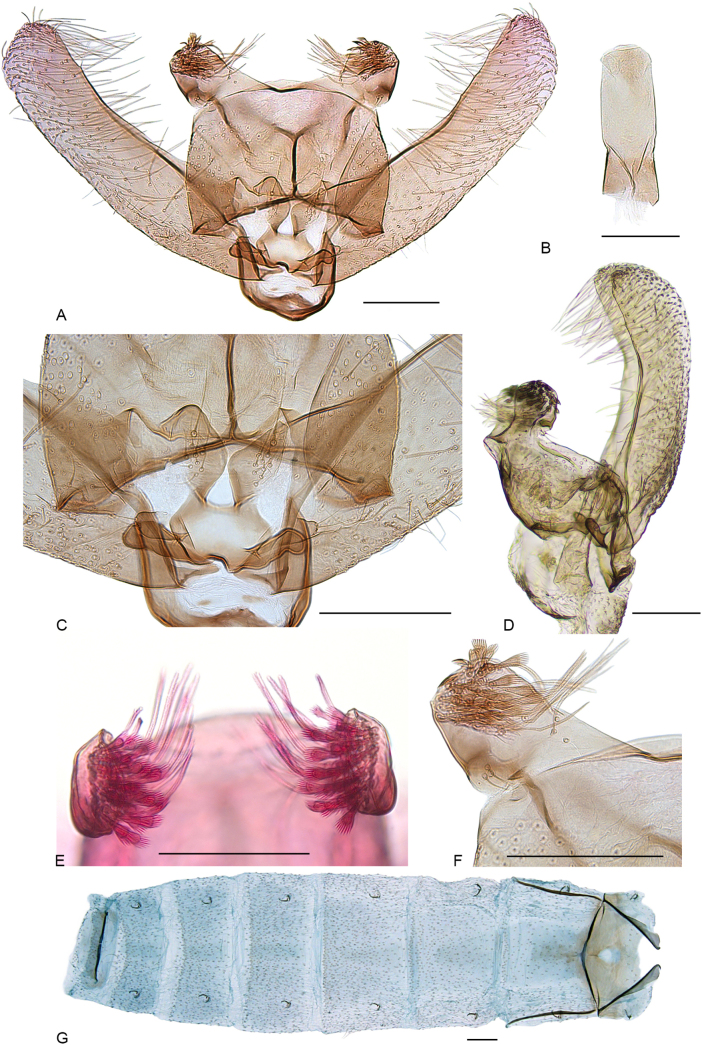
*Elachista agassizi* sp. nov., male genitalia. **A**. General view, phallus removed, holotype; **B**. Phallus, holotype; **C**. Juxta region, holotype; **D**. Lateral view, paratype, gen. prep. VS588; **E**. Uncus lobes, ventro-distal view, paratype, gen. prep. VS588; **F**. Uncus lobe, ventral view, holotype; **G**. Abdomen, holotype (**D**, **E** in glycerol before permanent mounting in Euparal). Scale bars: 0.1 mm.

**Male genitalia** (Fig. 2). Tegumen short and broad, latero-inner margin strongly sclerotised in posterior 2/5. Uncus lobe small, nearly as long as wide; incision between lobes very wide; latero-basal margin strongly sclerotised. Ventral surface of uncus lobe with two groups of scales: sparse, long scales in basal part; in distal part, scales dense, short, club-shaped, and apically conspicuously pectinate. Gnathos completely absent. Valva long and narrow, about 4.5 times as long as its widest point; sacculus weakly bent, distal margin of cucullus oblique; costa weakly convex before middle of the valva, distal fold of costa vestigial. Median plate of juxta with a pair of large, strongly sclerotised, finger-like, dorsally directed lateral pockets. Juxta lobes medium-sized; median margin straight, joining straight distal margin at slightly obtuse angle; distal margin straight; ventral surface with group of setae baso-laterally. Digitate process short, twice as long as wide, with few setae apically. Vinculum short, U-shaped, proximal margin dilated distally; without prominent saccus. Phallus short and wide, about 0.4× as long as the valva; apex blunt; vesica without cornuti.

#### Biology.

Unknown.

#### Flight period.

Adults were collected from late November until early December.

#### Distribution.

Central Kenya.

#### Etymology.

Named after David John Lawrence Agassiz, a British entomologist who collected the type specimens.

### Elachista knudlarseni sp. nov.

Taxon classificationAnimaliaChordarialesChordariaceae

E66F8AFC-3CB8-5C7A-9530-A17932F76F29

https://zoobank.org/CEFFA40E-1DFF-4E57-BEED-89178B3091BC

Figs 3, 4

#### Material examined.

***Holotype***. Tanzania • ♂; Iringa, Kigogo forest; 1880 m; 18 km S. Kibau; 26–28. Nov. 2004; K. Larsen & T. Zandersen leg.; gen. prep. VS398; VU.

#### Diagnosis.

*Elachista knudlarseni* belongs to the *E. bifasciella* species group. Superficially, *E. knudlarseni* resembles many species of the *E. bifasciella* species group, which have a white transverse fascia on a dark-coloured forewing. However, the male genitalia are highly distinctive, characterised by a narrow, parallel-sided cucullus with a sharply angled dorso-terminal margin and a tapered digitate process. Until now, only two species of the *E. bifasciella* species group were known from tropical Africa: *E. iriphaea* (Meyrick, 1932) from Uganda and *E. planca* Sruoga & De Prins, 2009 from Kenya. The male genitalia are known only for *E. iriphaea*, which, based on the characters described here, differs markedly from *E. knudlarseni*. *Elachista planca* is known only from adult and female genitalia, but this species is clearly larger, and its forewing is narrower than in *E. knudlarseni*.

#### Description.

**Figure 3.**
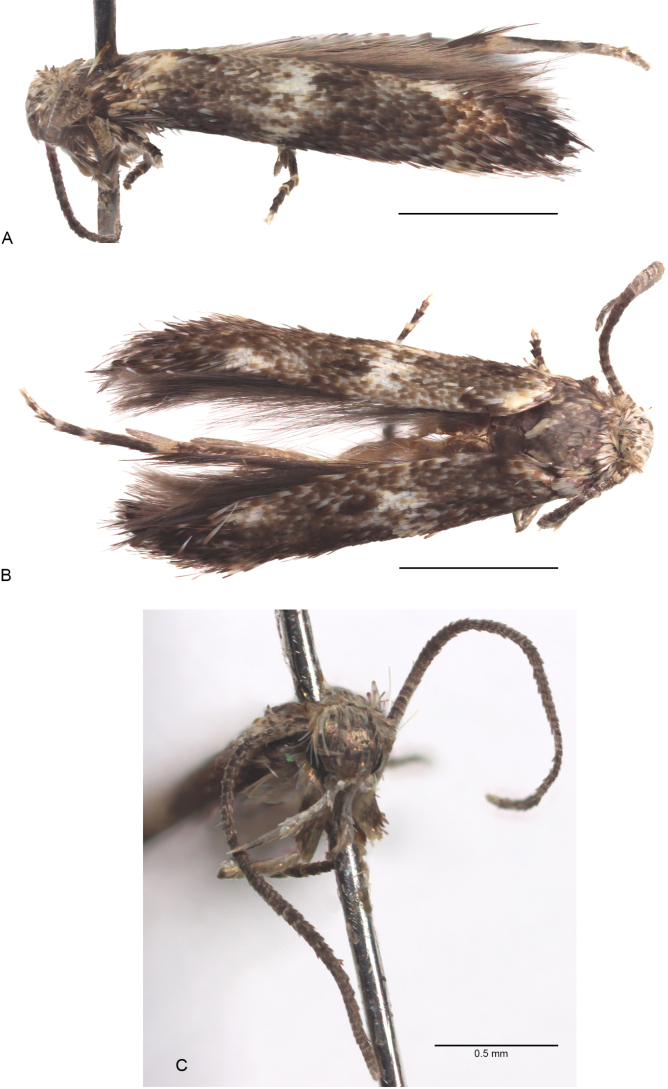
*Elachista knudlarseni* sp. nov., adult male, holotype. **A**. Lateral view; **B**. Dorsal view; **C**. Head, frontal view. Scale bars: 1 mm (**A, B**).

**Male**. (Fig. 3). Forewing length 3.1 mm; wingspan 6.7 mm (*N = 1*). ***Head***: frons, vertex and neck tuft greyish white, some scales with brown-grey tips; labial palpus twice as long as width of head, white above and brown-grey below; antenna 0.7× as long as forewing, brown-grey, except last flagellomere greyish white; flagellum weakly annulated in basal part. ***Thorax***, tegula, and forewing strongly mottled with scales, basally greyish white and distally greyish brown; antemedian transverse fascia greyish white, blurred; blackish brown scales forming two irregular spots beyond fascia; small costal and tornal spots blurred, greyish white; fringe greyish brown. Hindwing and its fringe greyish brown.

**Female**. Unknown.

**Figure 4.**
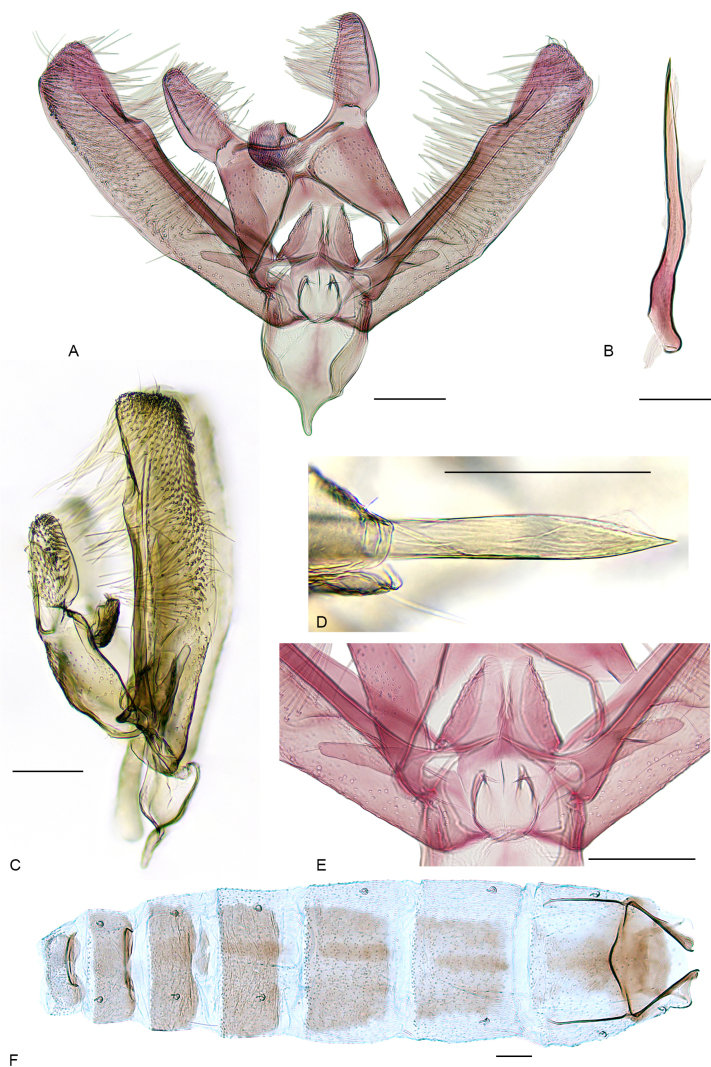
*Elachista knudlarseni* sp. nov., male genitalia, holotype. **A**. General view, phallus removed; **B**. Phallus; **C**. Lateral view; **D**. Distal part of phallus; **E**. Juxta region; **F**. Abdomen (**C**, **D**, in glycerol before permanent mounting in Euparal). Scale bars: 0.1 mm.

**Male genitalia** (Fig. 4). Uncus lobes narrow, as long as wide, tapered and widely separated by a U-shaped incision; median margin straight, lateral margin curved in basal part. Basal arms of gnathos reinforced; spinose knob slightly wider than long. Valva nearly straight; basal fold of costa extending to distal 3/4 of valva, where it meets distal lobe and forms broad, indistinct hump; cucullus parallel-sided, not produced dorsally, dorsal and terminal margins forming an angle of 70°. Juxta lobes large, median margin straight, joining straight distal margin at right angle, distal margin convex, ventral surface laterally with few setae. Digitate process slender and tapered, almost glabrous, with one or two setae apically. Vinculum with weak median ridge; saccus narrow. Phallus almost 4/5 as long as valva, narrow, bent in basal part and tapered to acute apex; vesica without cornuti.

#### Biology.

Unknown.

#### Flight period.

The only known specimen was captured in late November.

#### Distribution.

Central Tanzania.

#### Etymology.

The species is named after Knud Larsen, a Danish entomologist who collected the type specimen.

#### Remarks.

The head in the holotype is somewhat abraded; therefore, the description is approximate.

### Elachista riftella sp. nov.

Taxon classificationAnimaliaChordarialesChordariaceae

5F535597-7321-5E3F-8DE4-4206086B9E95

https://zoobank.org/CCF7CB3B-3F07-4ECA-B804-5E84BE6E3096

Figs 5, 6

#### Material examined.

***Holotype***. Kenya • ♂; Rift Valley, Prov. Molo; 7500 ft; 24. Oct. 1998; D. J. L. Agassiz; gen. prep. L. Kaila 849; DJLA. ***Paratypes***. Kenya • ♂; Rift Valley, Prov. Turi; 8000 ft; 13. Dec. 1998; D. J. L. Agassiz leg. • ♂; same data except date; 13. Jan. 1999; D. J. L. Agassiz leg.; gen. prep. VS587 • ♂; same data except date; 15. Mar. 2000; gen. prep. VS636; DJLA.

#### Diagnosis.

*Elachista riftella* belongs to the *E. freyerella* species group. The wing pattern of this medium-sized species is hardly distinguishable from many others of the *E. freyerella* species group. By its markedly concave sacculus, strong distal spine of the sacculus, and needle-shaped cornutus, *E. riftella* resembles several other species of the *E. freyerella* species group: *E. spiculifera* Meyrick, 1922, known from South India (for illustrations refer to Sruoga and Diškus 2006), *E. alacera* Kaila, 2011, known from Australia (for illustrations refer to Kaila 2011), and *E. inscia* Meyrick, 1913, known from South Africa (for illustrations refer to Parenti 1988). The main differences are the following: (1) in *E. riftella*, the digitate process is evenly dilated apically, and the saccus is narrow, whereas in *E. spiculifera*, the digitate process is abruptly dilated apically, and the saccus is wider; (2) in *E. riftella*, the sacculus is strongly concave, the spinose knob of gnathos is small, and the vesica lacks spinules, whereas in *E. alacera* the sacculus is less concave, the spinose knob of gnathos is smaller, and small spinules are present in the vesica; (3) in *E. riftella*, the forewing is darkly coloured, and the spinose knob of gnathos is small, whereas in *E. inscia* the forewing is lightly coloured, and the spinose knob of gnathos is oval and very large.

#### Description.

**Figure 5.**
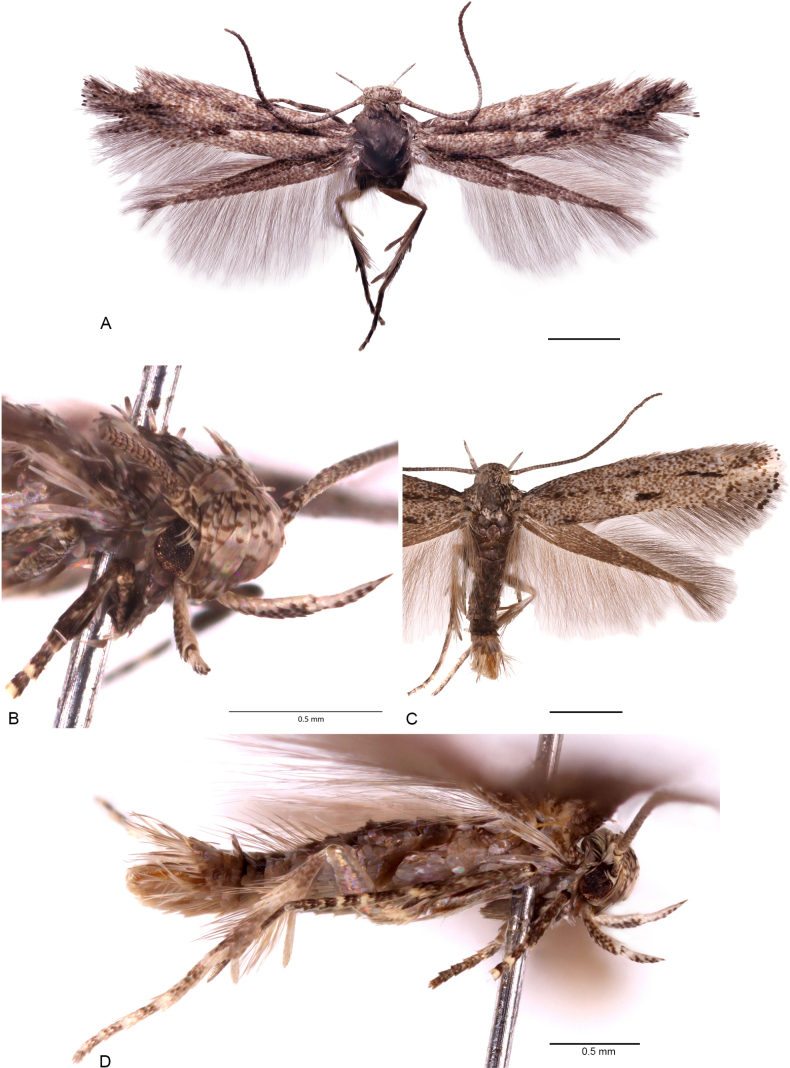
*Elachista riftella* sp. nov., adult male. **A**. Holotype; **B**. Head, fronto-lateral view, paratype; **C**. Paratype; **D**. Lateral view, paratype. Scale bars: 1 mm (**A**, **C**).

**Male**. (Fig. 5). Forewing length 3.0–4.1 mm; wingspan 6.7–8.6 mm (*N = 4*). Head: frons creamy white; lowermost scale layer of clypeal area more or less brownish grey; vertex and neck tuft greyish white, with greyish brown tipped scales; labial palpus upwards curved, diverging, about 1.5× as long as width of head, creamy white above, greyish brown below; antenna 0.7 as long as forewing, scape creamy white, intermixed with greyish brown, flagellum brownish grey, without annulation; pecten present as a few short, creamy white seta-like scales at base of scape. Thorax and tegula creamy white, intermixed with greyish brown. Forewing composed of basally creamy white and distally light-brown tipped scales, giving mottled appearance; row of blackish brown scales along fold extends to 0.4 of forewing; small, elongated blackish brown spot is present at dorsal margin in basal 1/6; plical spot elongate, blackish brown, at ½ wing length on dorsal side of fold; similar but smaller opposite spot near costal margin; blackish brown discal spot forms long streak at ⅔ wing length in middle; costal and tornal spots indistinctly delimited; fringe scales brownish grey, fringe line blackish brown. Hindwing grey-brown, its fringe scales somewhat paler.

**Female**. Unknown.

**Figure 6.**
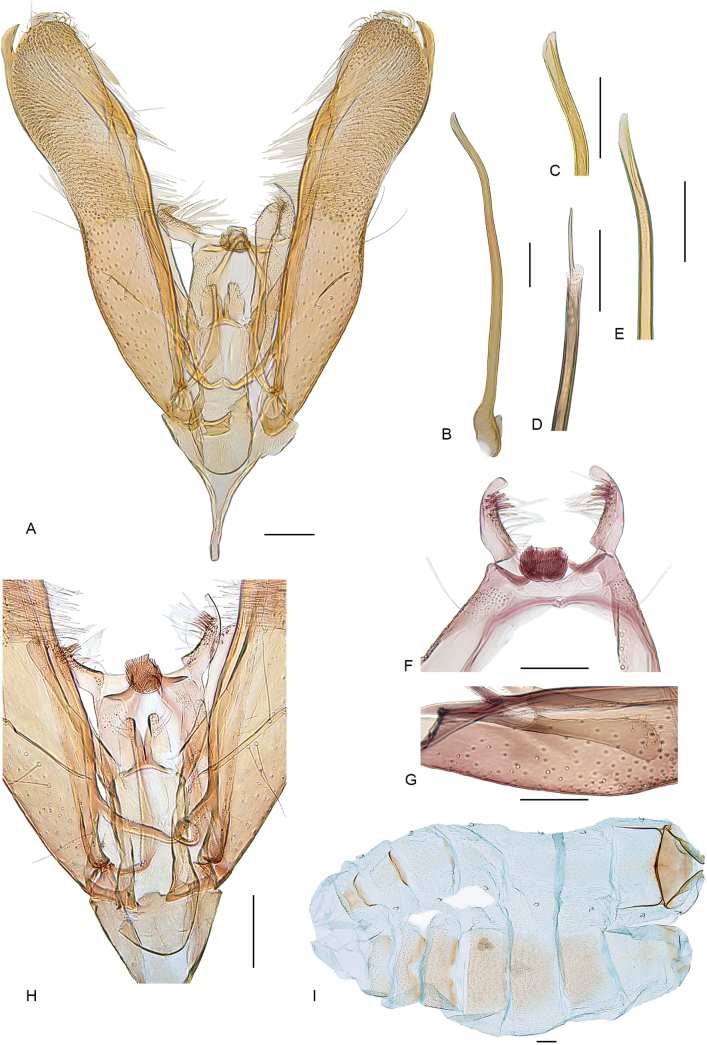
*Elachista riftella* sp. nov., male genitalia. **A**. General view, phallus removed, holotype; **B**. Phallus, holotype; **C**. Idem, distal part; **D**. Idem, paratype, gen. prep. VS587 (in glycerol before permanent mounting in Euparal); **E**. Idem, paratype, gen. prep. VS636; **F**. Uncus and gnathos, paratype, gen. prep. VS587; **G**. Digitate process, paratype, gen. prep. VS587; **H**. Central part, paratype, gen. prep. VS636; **I**. Abdomen. Scale bars: 0.1 mm.

**Male genitalia** (Fig. 6). Uncus lobes narrow and long, evenly curved and tapered to pointed apex; ventral surface covered with mixture of short and thick, and long and slender scales. Basal arms of gnathos short and reinforced, spinose knob small, nearly rounded. Valva broadest in basal part; sacculus strongly concave medially, distally with stout spine; cucullus neither expanded nor produced towards costa, apex nearly rounded; basal fold of costa extended to ⅔ of valva, where it meets distal fold forming broad indistinct hump. Median plate of juxta 3 times as long as broad, proximal end formed as rounded sac; medial margin of juxta lobes strongly sclerotised; lobes elongate, broadest medially, tapered distally; ventral surface with few long and short setae. Digitate process 0.44× as long as valva, narrow, distally somewhat dilated and setose. Vinculum produced into long, narrow, blunt-tipped saccus. Phallus about 0.9× as long of valva, narrow, broadest basally, curved at distal 1/5; caecum dorsally with cusp-like lobe whose anterior margin is concave; vesica with one needle-like cornutus, about 1/8 length of phallus.

#### Biology.

Unknown.

#### Flight period.

Probably two generations per year. Moths were collected from late October until mid-January and in mid-March.

#### Distribution.

Western part of Kenya.

#### Etymology.

The species name is derived from the word “rift”, as in Rift Valley.

## Discussion

All three new species described herein exhibit striking, clearly distinctive morphological differences in their male genitalia. *Elachista agassizi* sp. nov. is the most distinctive, characterised by a completely reduced gnathos. This condition is, so far, unique within Elachistinae. The complete absence of a spinose knob on the gnathos is also very rare and has been reported only in three *Elachista* species of the subgenus *Hemiprosopa* Braun. However, several other characters of the male genitalia of *E. agassizi* sp. nov. do not conform to the diagnostic features of this subgenus.

Partial reduction of the spinose knob of the gnathos was recently reported in *E. conica*Sruoga and Kaila (2026), which belongs to the *E. praelineata* species group of the subgenus *Elachista* Treitschke. *Elachista agassizi* sp. nov. somewhat resembles a few members of the *E. praelineata* species group in lacking a distal fold of the valva and in possessing large lateral pockets on the juxta; however, its placement within this or any other species group remains uncertain because it does not fully correspond to the key diagnostic features.

Including the species described herein, the genus *Elachista* now comprises 25 species in sub-Saharan Africa. The highest diversity is recorded in South Africa (8 species) and Kenya (8 species), while 1–4 species are known from each of seven other African countries, including Saint Helena and Madagascar. Based on type localities and available records, most species are currently documented from only one or two countries. Given the region’s biological richness, this species count is clearly an underestimate and does not reflect the true diversity of the genus. Large areas of sub-Saharan Africa remain unexplored for *Elachista*, indicating that further targeted collecting will likely reveal many additional species.

### Checklist of the *Elachista* species of sub-Saharan Africa

**1. *Elachista agassizi* sp. nov**.

**Distribution**. Kenya.

**Holotype**. ♂ and 3 paratypes ♂♂ in DJLA.

**2. *Elachista brevis* Sruoga & De Prins, 2009**

*Elachista brevis*Sruoga and De Prins 2009: 4; Kaila 2019: 31.

**Distribution**. Kenya.

**Holotype**. ♂ in RMCA.

**3. *Elachista chelonitis* Meyrick, 1909**

*Elachista chelonitis*Meyrick 1909: 25; Sruoga and De Prins 2009: 7; Kaila 2019: 36.

*Cleroptila chelonitis* (Meyrick); Meyrick 1914a: 204; Vári and Kroon 1986: 21; Parenti 1988: 190; Vári et al. 2002: 40.

**Distribution**. South Africa, Malawi, and Kenya.

**Holotype**. ♂ in TMSA.

**4. *Elachista conica* Sruoga & Kaila, 2026**

*Elachista conica*Sruoga and Kaila 2026: 10.

**Distribution**. Ethiopia.

**Holotype**. ♂ in MZH.

**5. *Elachista cordata* Sruoga & De Prins, 2011**

*Elachista cordata*Sruoga and De Prins 2011: 2; Kaila 2019: 42.

**Distribution**. Cameroon.

**Holotype**. ♂ in RMCA.

**6. *Elachista crocogastra* Meyrick, 1908**

*Elachista crocogastra*Meyrick 1908: 733; Vári and Kroon 1986: 25; Vári et al. 2002: 40; Sruoga 2002: 140; Kemal and Koçak 2006: 4; Parenti 2006: 142; Kaila 2019: 43; Lees and Minet 2022: 1147.

**Distribution**. South Africa and Madagascar.

**Syntypes**. Four syntypes in NHMUK.

**7. *Elachista delocharis* Meyrick, 1932**

*Elachista delocharis*Meyrick 1932a: 115; Kaila 1999: 167; Vári et al. 2002: 40; Sruoga 2002: 138; Kaila 2019: 46.

**Distribution**. Ethiopia.

**Holotype**. ♀ in NHMUK.

**8. *Elachista griseifrons* Sruoga & Kaila, 2026**

*Elachista griseifrons*Sruoga and Kaila 2026: 6.

**Distribution**. Ethiopia.

**Holotype**. ♂ in MZH.

**9. *Elachista gypsophila* Meyrick, 1911**

*Elachista gypsophila*Meyrick 1911: 233; Vári and Kroon 1986: 40; Parenti 1988: 190; Kaila 1999: 165; Vári et al. 2002: 40; Sruoga 2002: 140, Kemal and Koçak 2006: 4; Kaila 2019: 69.

**Distribution**. South Africa.

**Lectotype**. ♂ and paralectotype ♀ in TMSA.

**10. *Elachista inscia* (Meyrick, 1913)**

*Mendesia inscia*Meyrick 1913: 322; Vári and Kroon 1986: 45; Vári et al. 2002: 40; Kemal and Koçak 2006: 4; Kaila 2019: 76.

*Cosmiotes inscia* (Meyrick); Parenti 1988: 191.

*Elachista inscia* (Meyrick); Kaila 1999: 168; Sruoga 2002: 140.

**Distribution**. South Africa.

**Lectotype**. ♂ and 1 ♂ paralectotype designated by Parenti (1988) (2 specimens from the type series not examined) in TMSA.

**11. *Elachista iriphaea* (Meyrick, 1932)**

*Labdia iriphaea*Meyrick 1932b: 213.

*Elachista iriphaea* (Meyrick); Sinev 2002: 164; Kaila 2019: 77.

**Distribution**. Uganda.

**Holotype**. ♂ in NHMUK.

**12. *Elachista justificata* Meyrick, 1926**

*Elachista justificata*Meyrick 1926: 340; Vári and Kroon 1986: 47; Parenti 1988: 190; Vári et al. 2002: 40; Sruoga 2002: 140; Kemal and Koçak 2006: 4; Kaila 2019: 79.

**Distribution**. South Africa.

**Holotype**. ♂ in SAMC.

**13. *Elachista kakamegensis* Sruoga & De Prins, 2009**

*Elachista kakamegensis*Sruoga and De Prins 2009: 8; Kaila 2019: 79.

**Distribution**. Kenya.

**Holotype**. ♂ and 2♀♀ paratypes in RMCA.

**14. *Elachista knudlarseni* sp. nov**.

**Distribution**. Tanzania.

**Holotype**. ♂ in VU.

**15. *Elachista levis* Sruoga & Kaila, 2026**

*Elachista levis*Sruoga and Kaila 2026: 8.

**Distribution**. Tanzania.

**Holotype**. ♂ in ZMUC.

**16. *Elachista longispina* Sruoga & De Prins, 2009**

*Elachista longispina*Sruoga and De Prins 2009: 11; Kaila 2019: 86.

**Distribution**. Kenya.

**Holotype**. ♂ in RMCA.

**17. *Elachista merimnaea* Meyrick, 1920**

*Elachista merimnaea*Meyrick 1920: 297; Parenti 1988: 189; Kaila 2019: 92.

**Distribution**. South Africa.

**Holotype**. ♂ in the SAMC.

**18. *Elachista merinaella* (Paulian & Viette, 1956)**

*Antispila merinaella*Paulian and Viette 1956: 145; Nieukerken and Geertsema 2015: 43.

*Antispila merinaella* Viette; Viette 1990: 25; Lopez-Vaamonde et al. 2019: 118.

*Elachista merinaella* (Viette); Lees and Minet 2022: 1147.

**Distribution**. Madagascar.

**Holotype**. ♀ in MNHN.

**19. *Elachista nymphaea* Meyrick, 1911**

*Elachista nymphaea*Meyrick 1911: 233; Vári and Kroon 1986: 61; Kaila 1999: 168; Vári et al. 2002: 40; Sruoga 2002: 140; Kemal and Koçak 2006: 4; Kaila 2019: 99.

*Cosmiotes nymphaea* (Meyrick); Parenti 1988: 191.

**Distribution**. South Africa.

**Lectotype**. ♀ designated by Parenti (1988) (one specimen from the type series not examined) in TMSA.

**20. *Elachista planca* Sruoga & De Prins, 2009**

*Elachista planca*Sruoga and De Prins 2009: 12; Kaila 2019: 109.

**Distribution**. Kenya.

**Holotype**. ♀ in RMCA.

**21. *Elachista riftella* sp. nov**.

**Distribution**. Kenya.

**Holotype**. ♂ and 3 paratypes ♂♂ in DJLA.

**22. *Elachista semophanta* Meyrick, 1914**

*Elachista semophanta*Meyrick 1914b: 281; Sruoga 2002: 138; Kaila 2019: 120.

**Distribution**. Malawi.

**Holotype**. ♂ in NHMUK.

**23. *Elachista silfverbergi* Sruoga & Kaila, 2026**

*Elachista silfverbergi*Sruoga and Kaila 2026: 4.

**Distribution**. Ethiopia and Kenya.

**Holotype**. ♂ and 40 ♂♂ paratypes in MZH; 1 ♂ paratype in DJLA.

**24. *Elachista sparsula* Meyrick, 1921**

*Elachista sparsula*Meyrick 1921: 114; Vári and Kroon 1986: 82; Parenti 1988: 189; Kaila 1999: 166; Vári et al. 2002: 40; Sruoga 2002: 140; Kemal and Koçak 2006: 4; Kaila 2019: 123.

**Distribution**. South Africa.

**Lectotype**. ♂ and 2 ♂♂ paralectotypes designated by Parenti (1988) (3 specimens from the type series not examined) in TMSA.

**25. *Elachista trifasciata* (Wollaston, 1879)**

*Stagmatophora trifasciata*Wollaston 1879: 437.

*Elachista trifasciata* (Wollaston); Sinev 2002: 164; Kaila 2019: 134; Fowler and Karisch 2020: 28.

**Distribution**. Saint Helena.

**Syntypes**. (12) in NHMUK.

## Supplementary Material

XML Treatment for Elachista agassizi

XML Treatment for Elachista knudlarseni

XML Treatment for Elachista riftella

## Additional information

### Artificial Intelligence (AI) use

The authors accept full responsibility for the content of the manuscript, including the disclosure of any use of AI.

No AI tools were used in the preparation of this manuscript.

### Author ORCIDs

V. Sruoga https://orcid.org/0000-0002-5687-8299

J. De Prins https://orcid.org/0000-0001-7637-5755

### Data availability

All of the data that support the findings of this study are available in the main text.
